# Supplementary material for: Elevated blood pressure in high-fat diet-exposed low birthweight rat offspring is most likely caused by elevated glucocorticoid levels due to abnormal pituitary negative feedback
Source: PLoS One. 2020 Aug 27;15(8):e0238223. doi: 10.1371/journal.pone.0238223 (PMC7451543; doi:10.1371/journal.pone.0238223)
Supplement: S1 Table — (DOCX) [file pone.0238223.s001.docx]

S1 Table

Rodent diet with 40kcal% calorie restriction diet we used in this study

Low carbohydrate calorie restriction Standard chow

(LC)

gram kcal(%) gram kcal(%)

Protein 31.3 33 19.2 20

Carbohydrate 46.9 50 67.3 70

Fat 6.9 17 4.3 10

Total 100 100

Kcal/gram 3.75 3.85

Mineral Mix 10 10

S10026

Vitamin Mix 10 10

V10001

gram kcal gram kcal

Total 649.35 2434 1055.05 4057

Daily Food intake 212 354

(59.88%) (100%)
