# Supplementary material for: Elevated blood pressure in high-fat diet-exposed low birthweight rat offspring is most likely caused by elevated glucocorticoid levels due to abnormal pituitary negative feedback
Source: PLoS One. 2020 Aug 27;15(8):e0238223. doi: 10.1371/journal.pone.0238223 (PMC7451543; doi:10.1371/journal.pone.0238223)
Supplement: S1 Raw images — (PDF) [file pone.0238223.s003.pdf]

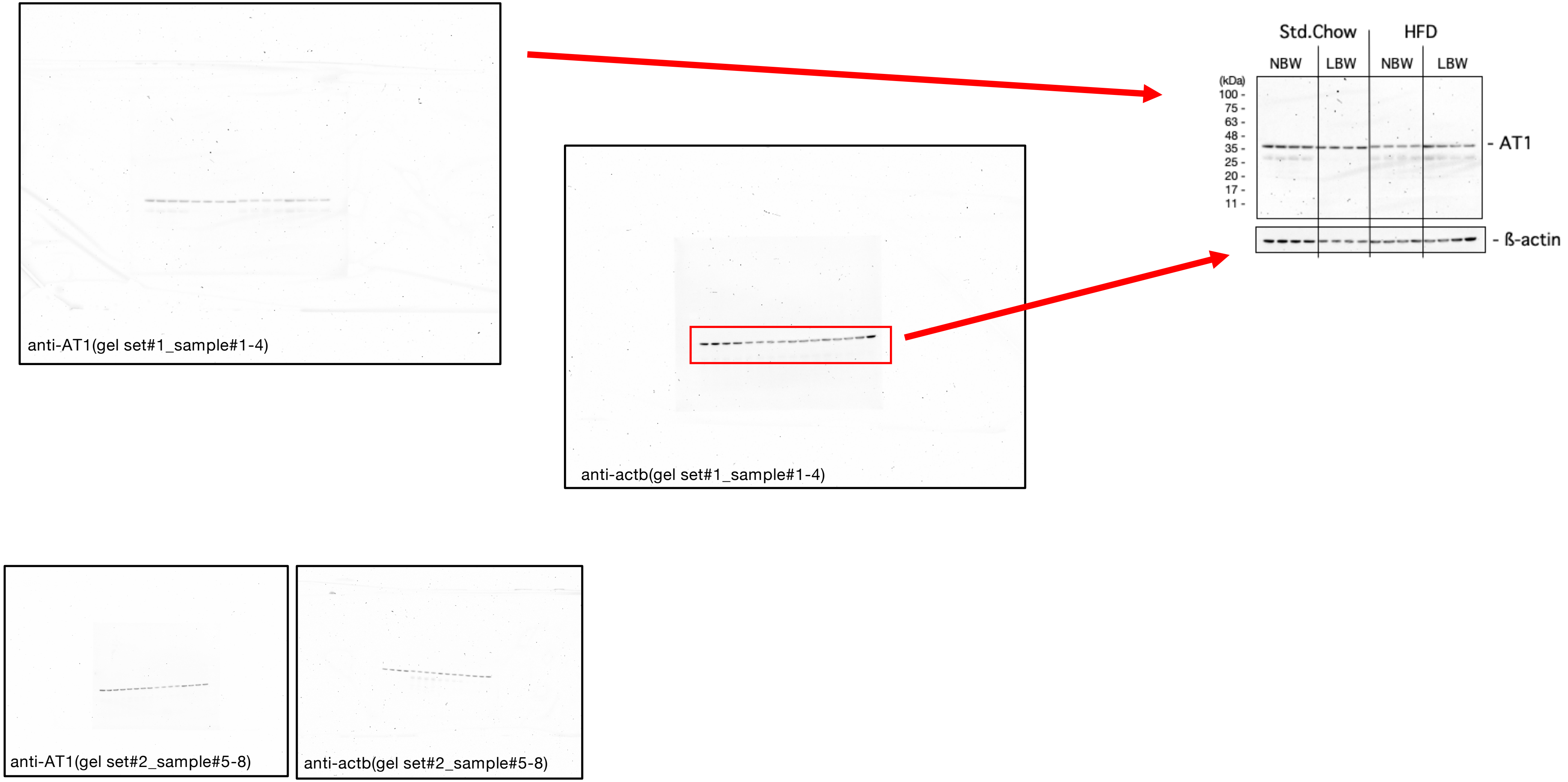


Raw Image of Figure 3C: Westernblot scan performed on a Bio-Rad ChemDoc system
Anti-AT1 antibody  (GeneTex , Cat.#GTX89149)
Anti-β-actin antibody (GeneScript, Cat.#A00730-40)

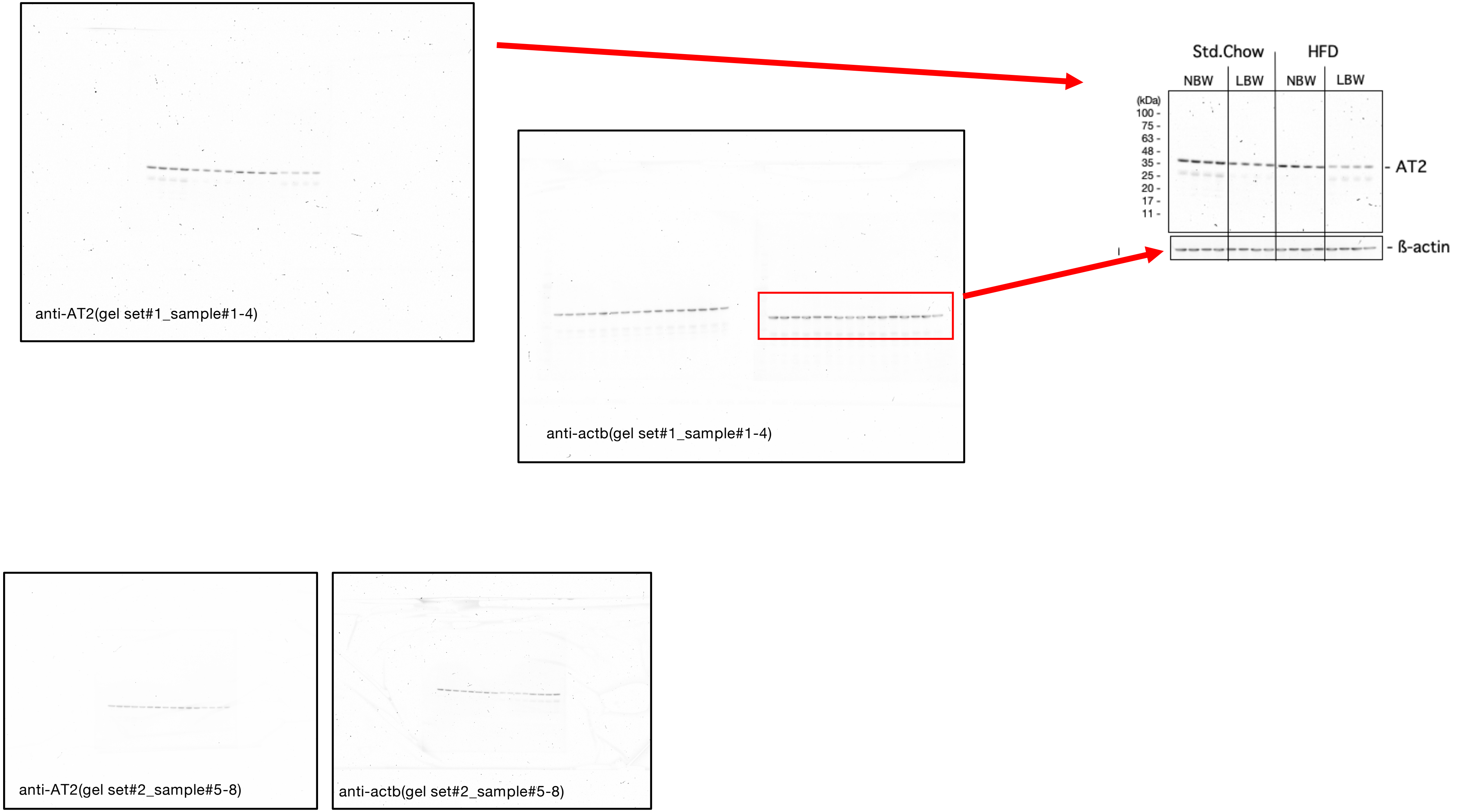


Raw Image of Figure 3D: Westernblot scan performed on a Bio-Rad ChemDoc system
Anti-AT2 antibody (GeneTex , GTX62361)
Anti-β-actin antibody (GeneScript, Cat.#A00730-40)

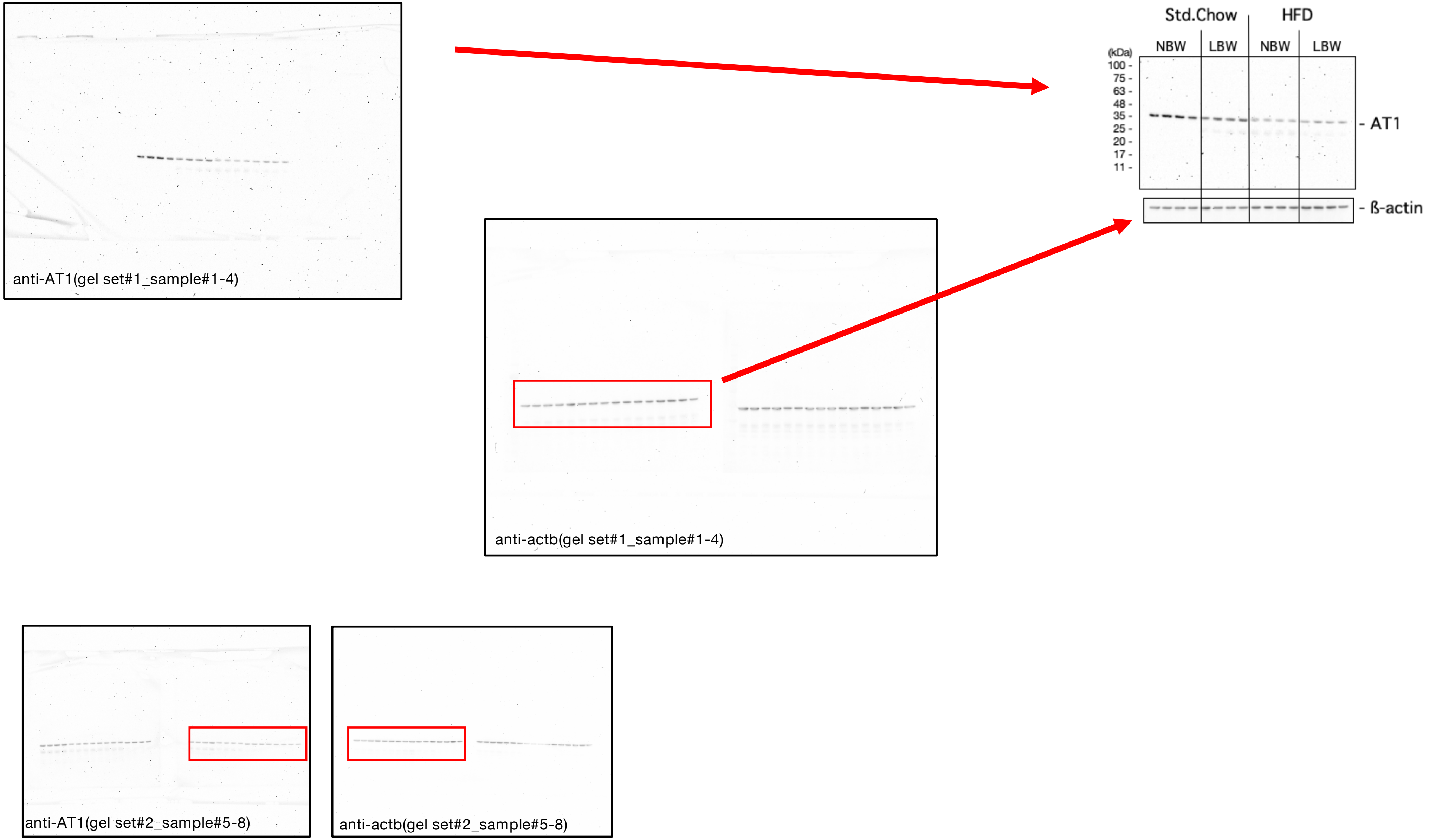


Raw Image of Figure 4C: Westernblot scan performed on a Bio-Rad ChemDoc system
Anti-AT1 antibody (GeneTex , Cat.#GTX89149)
Anti-β-actin antibody (GeneScript, Cat.#A00730-40)

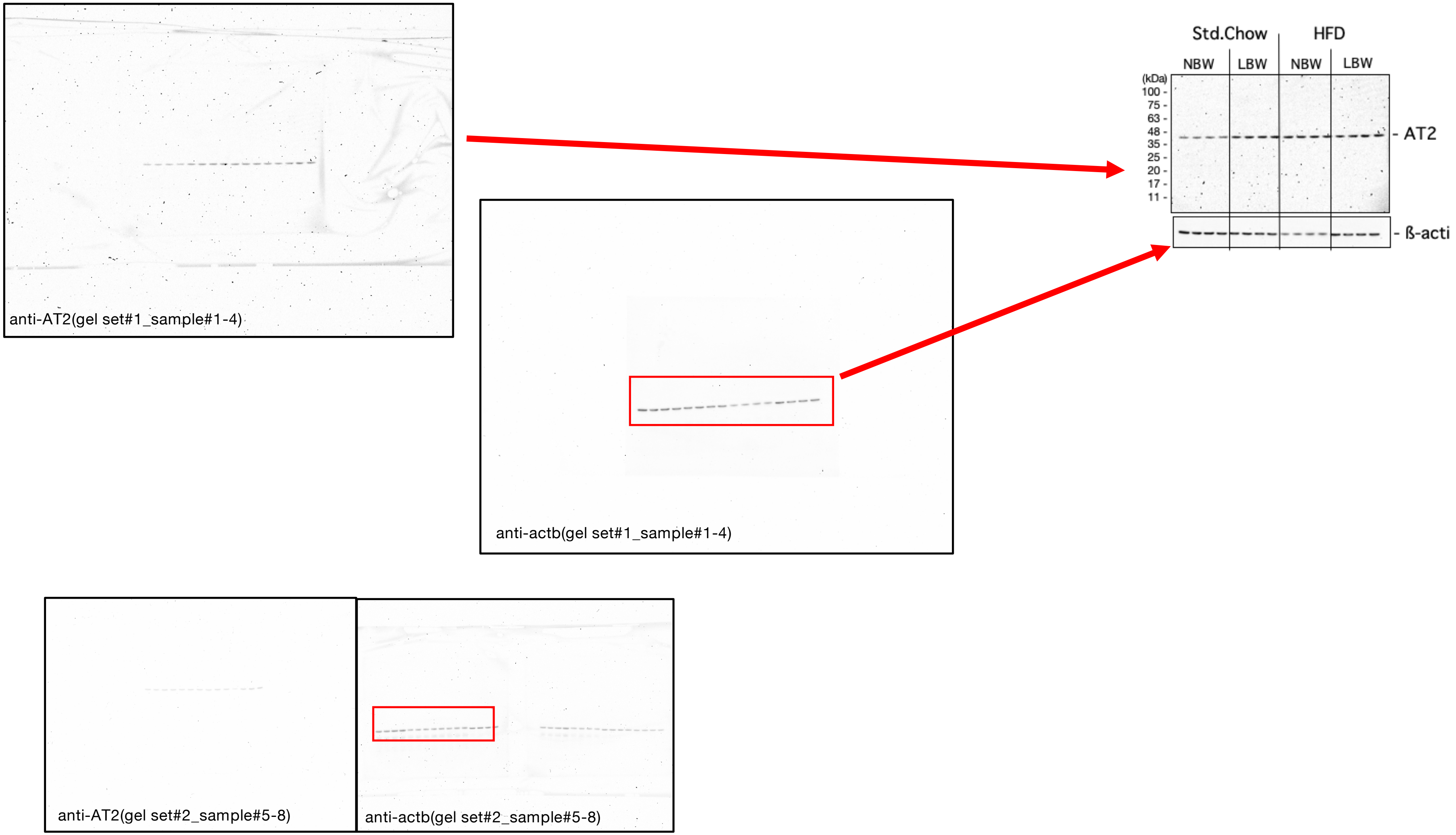


Raw Image of Figure 4D: Westernblot scan performed on a Bio-Rad ChemDoc system
Anti-AT2 antibody (GeneTex , GTX62361)
Anti-β-actin antibody (GeneScript, Cat.#A00730-40)
