# Supplementary material for: Elevated blood pressure in high-fat diet-exposed low birthweight rat offspring is most likely caused by elevated glucocorticoid levels due to abnormal pituitary negative feedback
Source: PLoS One. 2020 Aug 27;15(8):e0238223. doi: 10.1371/journal.pone.0238223 (PMC7451543; doi:10.1371/journal.pone.0238223)
Supplement: Attachment — Submitted filename: Rebutal_Letter_PONE-D-20-16514R1.docx [file pone.0238223.s005.docx]

Reviewer #1: The following concerns remain:

Weight at birth equals ~58 per group. This data should be presented as an average weight at birth per litter for the pups utilized for additional studies (excludes those culled).

Did average birth weight per litter for all viable pups (culled and utilized for additional studies) differ from average birth weight per litter for pups used for additional studies (excludes culled).

The weights of all offspring used in the experiment are shown in Supplementary table 2. Female rats were not used in the experiment, but there was no difference between the average weight including female rats and the average weight of male rats (used in the experiment).

Methods needs to describe on page 6 the number of pups separated at 5 weeks of age that are in each experimental and dietary group.

According to the reviewer’s comment, the number of rats was revised. We used control and LC offspring (46 rats each) divided into two groups (23 rats each); a standard diet and a high-fat diet.

A major concern is that after weaning, pups from all litters were mixed. Thus, can the authors ensure that only one pup per sex per litter was present in each study group versus multiple pups from the same litter included in the same dietary group. This is imperative in order to ensure a “programming” effect versus a “litter” effect.

We randomly mixed rat pups and used them in the experiment, but divided them so that each group contained one offspring from 10 dams. For example, the systolic blood pressure shows a mean blood pressure of 15 rats with 1 offspring from LC dam# 1- #5 plus 2 offspring from LC dam#6 - #10. The systolic blood pressure of a high fat diet exposed-offspring from LC dam #6 was 150.1 mmHg in one and 165.8 mmHg in the other. The high fat diet exposed-offspring from LC dam #7 was 146.1 mmHg on one and 154.0 mmHg on the other. They were higher than those of high fat diet exposed-control offspring. In other words, blood pressure does not increase only in the group born from a particular dam, and low birth weight rat pups due to fetal malnutrition tend to have increased blood pressure when exposed to a high-fat diet after birth. All data (including the dam ID) is available on figshare.com (<https://doi.org/10.6084/m9.figshare.12644165>).

Conclusions included on Page 11 within the first paragraph of the discussion that report an absence of pathological changes in the kidney and aorta (a non-resistance vessel) indicates a lack of involvement of renal and vascular disease as a component in the etiology of hypertension in this model is quite a stretch.

Conclusions that the mechanisms in this model differ from preterm infants is not unusual as this is not a model of preterm birth (line 287-288).

According to reviewer’s comment, we revised sentence in discussion section to “renal and vascular diseases may not play a causative role in the development of hypertension in our full-term fetal low-carbohydrate and calorie-restricted rat model.” (Changed to line 282-283)
